# Diffuse Alveolar Hemorrhage in Systemic Lupus Erythematosus: Histopathologic Features and Clinical Correlations

**DOI:** 10.1155/2017/1936282

**Published:** 2017-04-27

**Authors:** Robert Ta, Romulo Celli, A. Brian West

**Affiliations:** Department of Pathology, Yale University, 310 Cedar Street, New Haven, CT 06510, USA

## Abstract

The case of a 16-year-old African-American girl with systemic lupus erythematosus, who developed diffuse alveolar hemorrhage with fatal consequences, is described. Diffuse alveolar hemorrhage is a rare but serious complication of systemic lupus. It occurs in three distinct but overlapping phenotypes, acute capillaritis, bland pulmonary hemorrhage, and diffuse alveolar damage, each of which is associated with a different group of underlying conditions. Diffuse alveolar hemorrhage is a medical emergency: choice of treatment depends on early diagnosis and determination of the underlying etiology. Acute infection, superimposed on diffuse alveolar hemorrhage in the setting of immune compromise, is often a terminal event, as in this case.

## 1. Introduction

Diffuse alveolar hemorrhage (DAH) is a rare but serious complication of systemic lupus erythematosus (SLE) in children. It should be considered in any child with SLE who develops pulmonary infiltrates or has hemoptysis, hemosiderin-laden macrophages in sputum or in bronchoalveolar lavage, sudden drop in hemoglobin, thrombocytopenia, or signs of occult blood loss. It is associated with high mortality, often with systemic infection as a terminal event. The histology of DAH falls into three patterns, capillaritis, bland alveolar hemorrhage, and diffuse alveolar damage, each of which has its own differential diagnosis [1].

## 2. Clinical History

A 16-year-old African-American girl with a history of eczema and recent use of amoxicillin for swollen glands and a sore throat presented with unilateral periorbital edema. On questioning, it transpired that over the past three months she had experienced fatigue, night sweats, thirty-pound weight loss, and proximal muscle weakness. On examination, she had mild alopecia and focal mucosal ulceration. She had no joint pain or serositis. Significant findings on her initial blood work included anemia (hemoglobin: 9 g/dL) and elevated creatinine kinase (4840 U/L). The platelet count was in the normal range. She was admitted immediately to the pediatric unit. A rheumatological workup showed the following positive findings: ANA (titre > 1 : 2,560), anti-dsDNA, and anti-Smith. The patient was negative for anti-GBM, anti-Jo1, and SSA antibodies. The serum C3 levels were low and total IgG was elevated. In addition, repeated tests for the presence of antiphospholipid syndrome (phospholipid neutralization and Dilute Viper Venom Time assays) were negative. The overall findings were consistent with a diagnosis of systemic lupus erythematosus. The patient's weight loss, proximal muscle weakness, fatigue, and markedly elevated creatinine kinase were considered most likely due to a polymyositis overlap, but this possibility was never fully examined due to concern for the patient's rapidly worsening cardiac and pulmonary status. EMG, muscle imaging, or biopsy and serum rheumatoid assays were not performed.

The patient experienced episodes of tachycardia and hypertension raising concern for lupus myocarditis, but an EKG revealed only T wave inversions and echocardiography demonstrated good cardiac function. Troponin levels were normal initially but rose to a maximum of 5.16 ng/ml at about day 12 as cardiac function diminished and remained elevated. Echocardiography revealed overall good function and small pericardial effusion but was unable to rule out subclinical lupus myocarditis. Her cardiac function deteriorated as she developed premature ventricular contractions and runs of ventricular tachycardia, and repeat echocardiography demonstrated global dysfunction consistent with lupus myocarditis. She had episodes of severe hypotension that was unresponsive to fluids and vasopressors.

In addition to cardiac and pulmonary manifestations, the patient developed elevation of serum creatinine (to 1.0 mg/dl) and proteinuria (to 0.34 g/L). A percutaneous renal biopsy was performed to query the possibility of lupus nephritis. Histologic examination revealed mesangial proliferation, focal acute interstitial nephritis, and focal acute tubular injury. Other findings included mild interstitial scarring and a focal interstitial infiltrate of lymphocytes and plasma cells. Immunofluorescence studies demonstrated positive mesangial IgG, IgA, IgM, C3, and C1q staining. Electron microscopy revealed subepithelial, subendothelial, and mesangial electron-dense deposits with segmental effacement of foot processes. These overall findings were consistent with mesangial proliferative lupus nephritis (Renal Pathology Society class II) and concomitant acute tubular necrosis.

**Figure 1:**
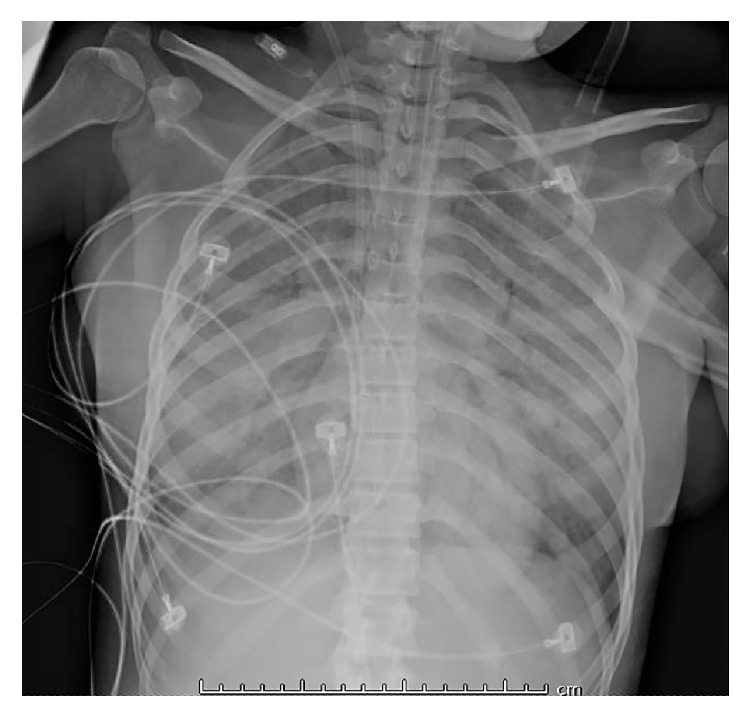
Chest radiograph showing diffuse dense bilateral airspace opacification with lower lobe predominance compatible with pulmonary hemorrhage/edema.

Immediately following her renal biopsy, the patient's oxygen saturation level dropped to 85% and she was placed on supplemental oxygen. Chest radiographs showed rapidly progressive bilateral pulmonary airspace opacification (Figure 1). This was associated with deterioration of respiratory function, and diffuse alveolar hemorrhage was suspected. Despite attempts to arrest her decline with plasmapheresis, intravenous immunoglobulin, cyclophosphamide, steroids, and rituximab, she died twenty-five days after admission.

## 3. Autopsy Findings

At autopsy, the principal findings were in the heart and lungs. There was no evidence of serositis.

**Figure 2:**
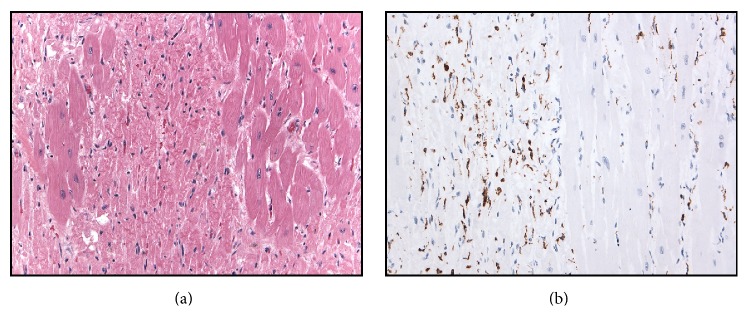
Myocardium of left ventricle. (a) H&E stain showing patches of normal appearing myocardium (right and left) separated by areas of confluent myocardial necrosis (center). (b) Immunostain for CD68 showing infiltrate of macrophages in necrotic areas (on left of field), with sparing of the intact myocardium.

The heart was enlarged (weight 330 g, expected 110–250 g). The coronary arteries were in usual distribution and, on serial sectioning at 0.5 cm intervals, showed <25% atherosclerotic stenosis. All four chambers and the tricuspid, pulmonary, mitral, and aortic valves were unremarkable, without evidence of endocarditis. The left ventricular free wall was 1 cm thick. A pale area of myocardium involving the posterior and lateral walls of the left ventricle proved, on histology, to be composed of numerous sharply defined geographic areas of diffuse myocyte necrosis without fibrosis (Figure 2(a)). Necrotic areas contained a dense infiltrate of CD68-positive macrophages (Figure 2(b)), rare CD3-positive T cells, and virtually no CD38-positive B cells. Adjacent myocardium showed prominent contraction band necrosis. There was no significant infiltrate of lymphocytes, plasma cells, neutrophils, or eosinophils to suggest a myocarditis, although the absence of lymphoid and polymorphonuclear cells may have been a consequence of immunosuppressive therapy. Hemosiderin was not detected in the areas of necrosis. She was not treated with any known cardiotoxic medications such as chloroquine derivatives. These findings were interpreted as recent nonischemic lupus myocarditis with superimposed therapy effect.

**Figure 3:**
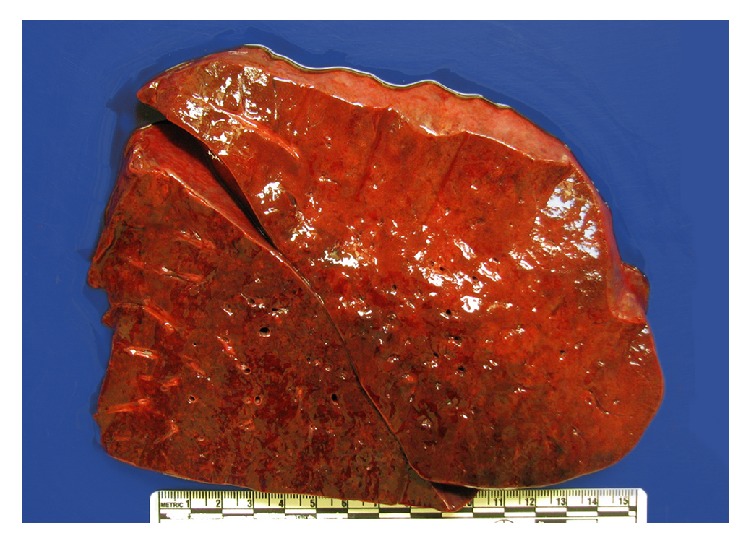
Gross photo of lung cut surface. The lung was solid, devoid of the usual floppiness.

**Figure 4:**
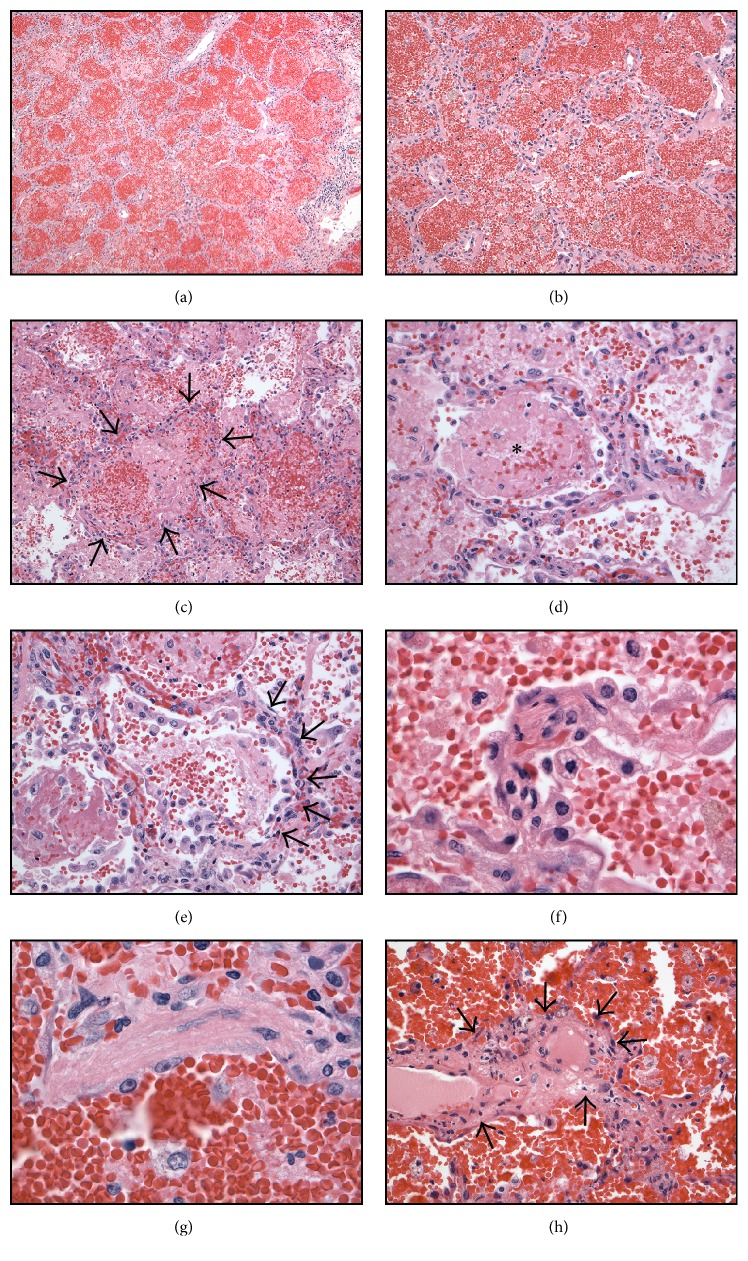
Lung with diffuse alveolar hemorrhage (H&E stain). (a) Area in which all alveoli are filled with extravasated erythrocytes. (b) Higher power view of same region. Note the hypercellularity of the alveolar septa. (c) A mixture of red cells and fibrin predominates in some alveoli (arrows). (d) The central alveolus contains a fibrin plug (asterisk). The alveolar septum is hypercellular. (e) The alveolar septum shows capillaritis (arrows), an inflammatory infiltrate containing neutrophils that degenerate releasing toxic compounds, causing capillary injury and leakage of erythrocytes. (f) High power view of septum with capillaritis. (g) A hyaline membrane abuts a damaged alveolar septum. (h) There were occasional foci of small vessel vasculitis (arrows).

The lungs were heavy (left 940 g, expected 170–250 g; right 860 g, expected 200–300 g). The parenchyma was firm, red, and congested and oozed bloody fluid from freshly cut surfaces (Figure 3). Histology revealed filling of alveoli by red cells in much of the lung parenchyma (Figures 4(a) and 4(b)) and in some regions by fibrin plugs (Figures 4(c) and 4(d)). There was extensive capillaritis with invasion of the alveolar septa by inflammatory cells, notably neutrophils (Figures 4(e) and 4(f)). Hyaline membranes were present focally (Figure 4(g)). Rare foci of small vessel vasculitis were present (Figure 4(h)), but intravascular thrombi and acute or chronic bronchopneumonia were not seen.

**Figure 5:**
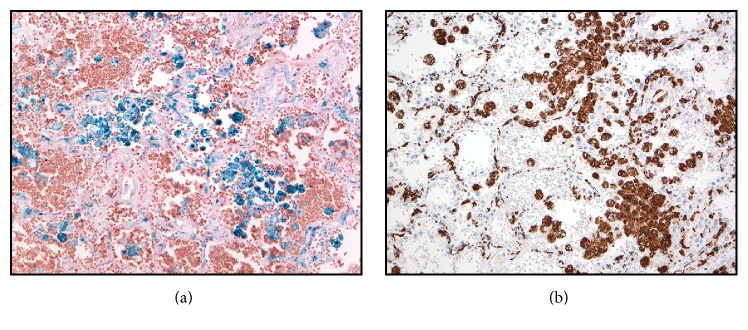
Area of lung with abundant hemosiderin-laden macrophages, singly and in clusters. (a) Prussian Blue stain confirming the presence of hemosiderin (blue). (b) CD68 immunostain demonstrating that the cells are macrophages (brown).

There were numerous CD68-positive intra-alveolar histiocytes in areas forming dense clusters, many of which contained iron (Figures 5(a) and 5(b)). These features are typical of diffuse alveolar hemorrhage. The remaining organs of the complete autopsy were unremarkable.

Postmortem cultures of lung and blood were positive for* Pseudomonas aeruginosa*, as was a premortem urine culture taken on day 18 of admission.

The cause of death was systematic lupus erythematosus involving multiple organs, manifested by diffuse alveolar hemorrhage, nonischemic cardiac necrosis, and lupus nephritis, complicated by* Pseudomonas aeruginosa* sepsis.

## 4. Discussion

### 4.1. Clinical Features of Patients with Juvenile Onset Systemic Lupus Erythematosus

Araujo et al. [2] found that patients with juvenile onset SLE had a higher frequency of alveolar hemorrhage compared to patients with adult onset SLE. Alveolar hemorrhage was the first manifestation of SLE in two juvenile SLE cases. Similar symptomatic findings included hemoptysis, dyspnea, tachycardia, hypoxemia, and pulmonary infiltrates on chest X-ray. Extrapulmonary manifestations of SLE included nephritis (63.6%), cutaneous disease (27.3%), arthritis (18.2%), serositis (27.3%), and psychosis (9.1%). Thrombocytopenia was found in 81.8% of patients. In terms of treatment, a higher percentage of juvenile onset SLE patients were treated with intravenous immunoglobulin (39%), plasmapheresis (20%), and cyclophosphamide (69%). From a critical care perspective, there was a high percentage of mechanical ventilation (85%), sepsis (50%), and ultimately death (69.2%) in the juvenile onset group.

### 4.2. Diffuse Alveolar Hemorrhage and Systemic Lupus Erythematosus

Pulmonary hemorrhage is a rare but serious complication of SLE in children. Acute pulmonary hemorrhage is one of the most severe forms of pulmonary involvement and is said to occur in less than 5% of children with SLE: in one study of 410 children with SLE from a pediatric tertiary hospital, only 7 had DAH (1.7%) (Singla et al.) [3]. Nevertheless, any pediatric patient with SLE who has acute shortness of breath along with a sudden drop in hemoglobin should be assessed for acute pulmonary hemorrhage. It is also possible for some children to have asymptomatic chronic intrapulmonary bleeding that may come to attention because of the presence of hemosiderin-laden macrophages in the sputum. In addition, those with chronic pulmonary hemorrhage may simply present with anemia. Other clues may include an elevated reticulocyte count with a negative Coombs test. Chang et al. [4] found that the most common presentation was dyspnea and fever followed by hemoptysis, but in their experience Martínez-Martínez and Abud-Mendoza [5] found that hemoptysis was a less common presentation. Thrombocytopenia may also suggest DAH. In a study of 1,000 patients with SLE of whom 22 had DAH, Kazzaz et al. [6] found that thrombocytopenia was predictive of DAH with an odds ratio of 40.

### 4.3. Systemic Infection in Patients with SLE

Patients with SLE are at increased susceptibility to infection because of both their SLE-related immune dysregulation and their treatment with immunosuppressive regimens, and infections are among the most common complications in children with SLE [7]. In 24 patients who died with SLE-associated DAH, Martinez-Martinez et al. [8] found that 22 died from infection. Faco et al. [9] reported that patients with juvenile SLE had* Staphylococcus aureus* as the most common infection (50%), with* Pseudomonas aeruginosa* being the second most common infection (17%). Martinez-Martinez et al. [8] reported similar results with 8/16 bacterial infections due to* S. aureus* and 3/16 due to* P. aeruginosa.* Since our patient was given immunosuppressive drugs and had active SLE with evidence of hypocomplementemia, it is not surprising that she developed infection with one of these nosocomial pathogens. In this instance, whether* Pseudomonas *was a cause of capillaritis and diffuse alveolar hemorrhage or a secondary invader is not clear.

### 4.4. Pathology of Diffuse Alveolar Hemorrhage

Diffuse alveolar hemorrhage occurs in three distinct but sometimes overlapping histologic patterns: pulmonary capillaritis, bland pulmonary hemorrhage, and diffuse alveolar damage [10].* Pulmonary capillaritis* is defined by neutrophil infiltration of the alveolar septa, loss of capillary structural integrity, and spilling of red blood cells into the alveolar spaces and interstitium [11]. Neutrophils degenerate and undergo fragmentation, releasing noxious by-products such as toxic oxygen radicals and proteolytic enzymes, causing additional injury to the capillaries and further alveolar hemorrhage. In some patients, pulmonary capillaritis leading to diffuse alveolar hemorrhage has been attributed to the presence of antiphospholipid antibodies [12], but this cannot have been the situation here as assays for antiphospholipid antibodies were negative.* Bland pulmonary hemorrhage* presents microscopically with accumulation of erythrocytes and fibrin in the alveolar spaces without inflammation or destruction of alveolar structures [13]. Lastly,* diffuse alveolar damage* is characterized by edema of alveolar septa and formation of hyaline membranes [14]. In the present case, the predominant pattern is that of pulmonary capillaritis. The main entities in the differential diagnosis of the three morphological forms of DAH are listed in Table 1.

### 4.5. Clinical Features of Diffuse Alveolar Hemorrhage

Diffuse alveolar hemorrhage may present with several signs and symptoms including cough, hemoptysis, fever, and dyspnea and with mild-to-severe forms of respiratory distress which usually follow a progressive course. Initial investigations with radiology and laboratory testing can be helpful in determining a cause for the alveolar hemorrhage. Because of the numerous other causes of cough, dyspnea, and respiratory distress, it is important in the first place to determine if the symptoms are due to alveolar hemorrhage rather than an alternative process. Once the diagnosis of DAH is established, effort should be focused on determining the nature of the underlying disease so that appropriate therapy can be instituted. Radiographic examination often shows patchy or diffuse opacities on chest X-ray (Figure 1). Computed tomographic scan of the chest can reveal ground glass or consolidative opacities diffusely and bilaterally. Laboratory abnormalities may include low hemoglobin or hematocrit, elevated white cell count, thrombocytopenia, and an elevated erythrocyte sedimentation rate (ESR).

## 5. Conclusion

Diffuse alveolar hemorrhage is a rare complication of SLE in children. The condition is associated with a high rate of mortality and requires early and complex management of both the underlying autoimmune process and the various complications. Early recognition and diagnosis therefore are of paramount importance in the management of patients with diffuse alveolar hemorrhage.

## Figures and Tables

**Table 1:** Selected differential diagnoses of the three histologic patterns of diffuse alveolar hemorrhage (after Park [1]).

Histologic patterns	Diseases
*Capillaritis*	

Systemic vasculitides	Behçet's syndrome
	Cryoglobulinemia
	Granulomatosis with polyangiitis
	Henoch- Schönlein purpura

Rheumatic diseases	Rheumatoid arthritis
	Systemic lupus erythematosus
	Antiphospholipid antibodies

Drugs	Hydralazine
	TNF-alpha antagonists

*Bland hemorrhage*	

Connective tissue disease	Goodpasture disease
	Systemic lupus erythematosus

Drugs	Anticoagulant therapy
	Platelet glycoprotein IIB/IIIA inhibitors

*Diffuse alveolar damage*	

Infection	Infection leading to ARDS (viral, bacterial)
	Opportunistic infections in compromised host

Rheumatic diseases	Polymyositis
	Systemic lupus erythematosus
